# Measuring unfinished nursing care in patients with or at risk for delirium: a development and Delphi study

**DOI:** 10.1007/s40520-026-03332-4

**Published:** 2026-03-10

**Authors:** Luisa Sist, Yari Longobucco, Rossella Messina, Stefania Chiappinotto, Paola Rucci, Alvisa Palese, UNCSD - Study Group, Andrea Buscaroli, Ester Palmieri, Alessandro Galazzi, Irene Mansutti, Maria Macchiarulo, Roberta Neri, Klara Komici, Nikita Valentina Ugenti, Laura Spessotti, Ermellina Zanetti

**Affiliations:** 1 Department of Biomedical and Neuromotor Sciences, Alma Mater Studiorum University of Bologna, Via Massarenti 9, Bologna, 40138 Italy; 2 IRCCS Azienda Ospedaliero-Universitaria di Bologna, Bologna, Italy; 3 Department of Health Sciences, University of Florence, Firenze, Italy; 4 Department of Medicine, University of Udine, Udine, Italy; 5 UNCSD (Unfinished Nursing Care Survey for Patients at Risk of and with Delirium) - Study Group, Bologna, Italy

**Keywords:** Delirium; Unfinished Nursing Care, Missed nursing care, Surveys and questionnaires, Delphi technique

## Abstract

**Background:**

Unfinished Nursing Care (UNC) refers to necessary patient care interventions that are delayed or omitted. Although several instruments exist to measure UNC, none are specifically designed for patients at risk of or experiencing delirium. These patients often have difficulty communicating their needs, making it harder for nurses to identify and prioritize appropriate care.

**Methods:**

A seven-round Delphi study, based on the Conducting and Reporting Delphi Studies (CREDES) framework, was conducted to develop the Unfinished Nursing Care Survey for Patients at Risk of and with Delirium (UNCSD) as an adaptation of the Unfinished Nursing Care Survey, composed of Part A and Part B. A multidisciplinary group evaluated the items using the Grading of Recommendations, Assessment, Development and Evaluation (GRADE) scale, which assigns scores from 1 to 9. The group reached consensus on the inclusion of items (70% agreement), with final consensus exceeding 90%. The Delphi process included three rounds for Part A, three for Part B, and one for the introduction section online, while one round was conducted in interactive mode to revise items and provide changes, mergers, additions, and exclusions.

**Results:**

The final UNCSD instrument consists of an introduction Section (31 items), Part A (39 essential nursing interventions, selected from an original set of 63), and Part B (23 reasons for UNC, reduced from 48).

**Conclusions:**

The UNCSD measures UNC and its reasons among patients at risk of or with delirium. Its use helps nurses identify gaps in care, improve decision-making, define care priorities, and ensure patient safety.

**Supplementary Information:**

The online version contains supplementary material available at 10.1007/s40520-026-03332-4.

## Introduction

Delirium is defined as a neuropsychiatric syndrome characterised by disturbances in attention (i.e., the ability to direct, focus, sustain, and shift attention is reduced), consciousness (i.e., orientation to the environment is reduced), and additional cognitive impairments (e.g., memory deficits, disorientation, language impairment, and visuospatial or perceptual difficulties). It typically occurs acutely and presents with fluctuating symptoms that represent a significant change from the patient’s baseline functioning [1]. Although available guidelines [2] recommend preventive interventions, delirium remains a significant clinical challenge not only because of its high prevalence—23.6% (95% CI: 19%–29%) among hospitalized older adults [3]—but also due to the variability observed across care settings, with rates ranging from 14.1% in rehabilitation facilities, 23.6% in hospitalized older patients, and 36.8% in nursing home residents [4], up to values between 12.5% and 83.9% in intensive care units. In addition, patients with or at risk of delirium are particularly vulnerable, as they are more likely to receive unfinished nursing care [5], which is associated with serious clinical consequences in the short term (e.g., increased incidence of hospital-acquired complications) [6] and long term (e.g., greater functional decline) [7].

The concept of unfinished nursing care (UNC) has been introduced in the literature in recent years [8]. It is an umbrella term that encompasses various conceptual models, terminologies, and tools that refer not only to Missed Nursing Care [9], but also to Implicit Rationing [10] and Tasks Undone [11]. UNC refers to all necessary nursing care measures that are either completely omitted or delayed [8]. This phenomenon has been analyzed using the socio-ecological model [12], which examines the problem from a complex, systemic perspective. This model identifies antecedents, such as the allocation of social resources (e.g., excessive workload and patient complexity [13]), and consequences, including compromised patient safety (e.g., deterioration of functional independence [14]) and reduced quality of care (e.g., patient dissatisfaction [15]).

When nurses omit or delay nursing care interventions in patients at risk of delirium, the probability of its occurrence increases; moreover, delaying some interventions may also prolong the persistence of delirium or worsen it. Sist et al. [16] documented in their review that out of 96 recommended delirium prevention or management interventions, only 35 are applicable and feasible in daily practice. The expected interventions are discussed in the literature as complex and multifaceted but often prove difficult to apply in daily clinical practice (e.g., minimizing the negative effects of the hospital environment) [17]. Therefore, not all recommended interventions are feasible, suggesting a higher risk of leaving them unfinished; on the other hand, targeted interventions addressing certain modifiable risk factors have been proven to significantly reduce the risk of delirium onset [18, 19]. As a result, patients at risk or with delirium require a high level of care that can be neglected [20]. Thus, measuring the occurrence of UNC can help nurses prioritize interventions aimed at preventing or reducing delirium, explain the different occurrences of delirium across settings, and address recent gaps highlighted in the literature that recommend developing tailored instruments capable of detecting issues in care delivered [21].

Several tools have been developed to assess UNC [22], among which the Unfinished Nursing Care Survey (UNCS) was designed and validated for general hospitalized patients (e.g [22–24])., to detect the phenomenon, identify its causes, and develop specific interventions to prevent or mitigate it [14]. Other tools have been validated [25], but none specifically in the context of delirium. Therefore, the aim of this study was to develop an instrument to measure UNC among at-risk patients and those with delirium through an innovative approach combining: (a) a deductive method, grounded in the available literature and best practice recommendations in this field [26], and (b) the tacit knowledge of experts regarding what works best in practice, operationalized through a Delphi technique [26].

## Methods

### Study design

The Delphi method was used to reach consensus in seven rounds according to the Conducting and Reporting Delphi Studies (CREDES) framework (Supplementary Table 1) [27] combined with a literature review [16, 28] to establish the Unfinished Nursing Care Survey for patients at risk and with Delirium (UNCSD). The Delphi procedure was conducted between June and September 2023.

#### Preparatory phase

To develop the tool, the researchers (see authors) first requested permission to use the UNCS tool from its author (Erika Bassi [23]). The tool was then adapted to the specific context of patients at risk and with delirium by revising the UNCS in the (1) general introductory section and the items in (2) Part A and (3) Part B as follows:

(1) The introduction section consists of 26 items derived from the UNCS tool [23]; additional items were proposed by the researchers (see authors), incorporating contributions from the available literature [12, 13, 29].

(2) Part A consists of 37 nursing care interventions from the UNCS tool: 35 interventions were derived from a systematic review of the literature conducted according to the Centre for Reviews and Dissemination [30] (January–February 2021) and the findings of the Nominal Group Technique [16]. After reviewing the items, the researchers (see authors) identified and grouped nine duplicate interventions.

(3) Part B is structured around 26 reasons from the UNCS instrument [23] and 22 from the integrative review conducted by the researchers according to Whittemore and Knafl’s framework and the Reporting Items for Systematic reviews and Meta-Analyses (PRISMA) guidelines [28].

Overall, after a process of addition (5 items in the introduction section), consolidation (9 items in Part A), and modification (4 items in Part B) by the researchers, the final version of the UNCSD instrument for the Delphi survey consisted of 31 items in the introduction section, 63 items in Part A, and 48 items in Part B.

#### Expert panel

The Delphi participants were experts selected for their experience with at-risk patients and patients with delirium. They came from both clinical and academic backgrounds and were required to have at least five years of professional experience and meet at least one of the following criteria: (a) in-depth knowledge of at-risk patients and those with delirium, acquired through research studies, research activities in the field, or academic teaching; (b) experience developing care pathways for at-risk patients and those with delirium, and managing the professionals involved in these pathways in clinical settings; or (c) recognized experience in caring for at-risk patients and those with delirium [31]. A panel of at least 10 experts as suggested by the literature [26] was established. The Delphi participants were invited via personalized email, which included detailed information about the study’s aim, participation requirements, and the informed consent form.

#### Consensus

First, the researchers decided to use the Grading of Recommendations, Assessment, Development and Evaluation (GRADE) scale [32] to assess the reliability of the items, as it allows consideration of the clinical relevance and applicability of the interventions (Part A) and the underlying reasons (Part B) in light of existing evidence, while facilitating maximum discrimination among items [32]. The consensus threshold was established a priori before the study began and was shared with a panel of experts [27, 33, 34].

Participants were asked to rate the importance of each item in Part A and Part B using a 9-point scale in line with the GRADE [32] methodology as follows: 1–3 (categorized as unimportant), 4–6 (important but not critical), and 7–9 (important and critical) [32]. A priori, overall average scores above 7 were considered, while scores below 5 were excluded, and scores between 5 and 6.9 were subject to reassessment [34]. For percentage agreement, the following thresholds were set: items with less than 50% agreement were excluded, items with 50–69% agreement were subjected to further evaluation, and items with 70% or more agreement were considered acceptable [27]. In the final stage, consensus was considered achieved if agreement was greater than or equal to 90% [35].

#### Delphi methods

The rounds were organized according to the structure of the instrument (introduction section, Part A, and Part B). Part A was addressed in the first three rounds, Part B in the next three rounds, and the general introduction in one round, resulting in a total of seven rounds. The process was conducted online using the Forms platform (Microsoft Corporation), and was preceded by an email outlining the aims and tasks for the round, a summary of previous results, the expected deadline, and instructions for the next steps. The second round included an online consensus meeting to ensure accessibility for the entire panel of experts, held via Teams (Microsoft Corporation), and dedicated to Part A.

The meeting was moderated by a group member (LS) and a supervisor (SC), who took notes. The session included: (a) a reminder of the study aims; (b) a description of the purpose and areas of consensus; (c) assessment of the intervention for inclusion or exclusion; and (d) discussion. All comments and amendments raised during the meeting were considered in subsequent rounds [27].

Part B participants who completed the GRADE assessment in the fourth round received a summary of their scores in table form and a new prompt to reassess using the same scale in the fifth round. They also received an assessment of the level of agreement based on the a priori criteria. Before assessing the level of agreement in the sixth round, the researchers sent participants a full summary of the results, including feedback and any written comments.

In the introductory section, participants assessed their level of agreement based on predefined criteria, approving the inclusion of five questions and the modification of three during the seventh round, while also providing additional written comments.

**Fig. 1:**
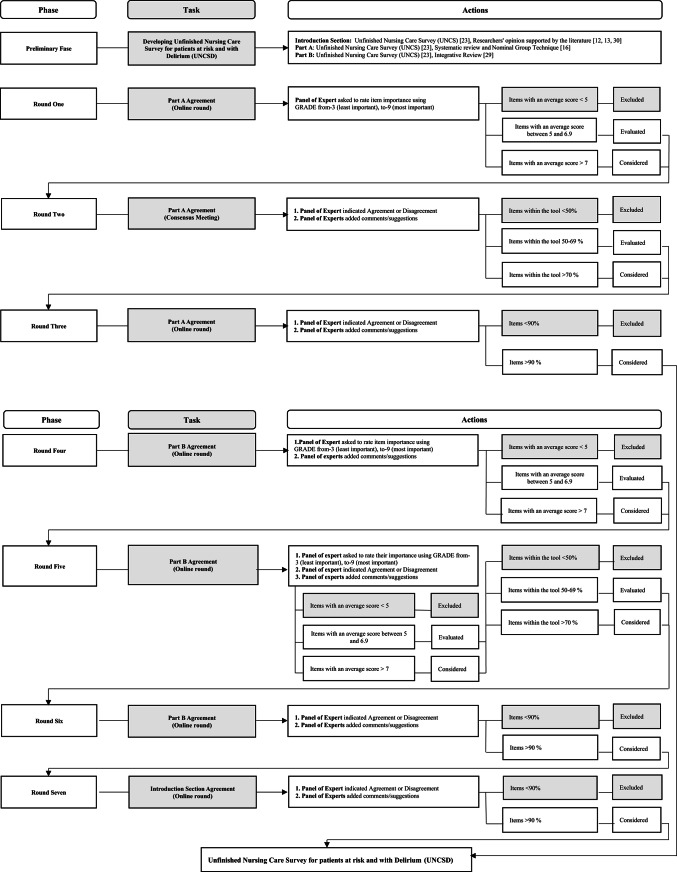
Flow chart illustrating the stages of the Delphi process, including the preparatory phase an all Delphi rounds

The final version of the instrument, the UNCSD, was then emailed to participants for member checking review [36], and their approval was sought via email response. Figure 1 illustrates the Delphi process.

### Data collection and analysis

The data was collected using an anonymous online tool that allowed the researchers to monitor and analyze the data. The online tool included the selected items and was amended in each subsequent round to incorporate responses from the previous round, with the aim of establishing consensus. Participants were also asked for personal details, including age, gender, education level, and field of work [33]. Each tool was left open for an average of ten days.

In each round, the panel of experts had the opportunity to add comments and suggestions in a free-text field to supplement, specify details, or provide justifications. All responses to open-ended questions were analyzed using content analysis [37]. Two researchers (SC and LS) performed the analysis manually, dividing it into three phases: (a) preparing the narratives; (b) reading and rereading the transcripts to identify units of meaning (relevant words or phrases); and (c) independently identifying sub-themes derived from the data, which were subsequently coded [37].

A descriptive analysis of the study variables was conducted using IBM SPSS Statistics Version 25.00 software.

### Ethical consideration

The study was conducted in accordance with the ethical standards of the Declaration of Helsinki [38] and was approved by the Bioethics Committee of the University of Bologna, Italy (No. 0151991, 6 June 2023). Participation by the expert panel was voluntary, and strict anonymity was guaranteed; they gave their consent to participate. Informed consent was also obtained from the participants in the consensus meeting.

## Results

### Expert panel

Ten experts participated in all rounds (100% response rate), with average age of that the average age is 41.5 years. Most were female (8; 80%) and held a master’s degree (4; 40%), a doctorate (3; 30%), or a medical specialization in geriatrics (2; 20%). At the time of participation, they worked as an advanced practice nurse (3; 30%), nurse manager (2; 20%), academic teacher of nursing (1; 10%), physician (1; 10%), or associate professor of medicine (1; 10%). 40% of participants had more than twenty years of experience; the overall average was 19 years (95% CI: 10.84–27.16) (Table  1).

**Table 1:** Characteristics of panel experts

Variables	*N* (%) 10 (100)
**Age, mean (95% CI)**	41.5 (32.89–50.11)
Professional experience, mean (95% CI)	19.0 (10.84–27.16.84.16)
5–9 years	3 (30)
10–19 years	3 (30)
>20 years	4 (40)
**Female**	8 (80)
**Education**	
Bachelor’s degree in nursing	1 (10)
Master’s degree	4 (40)
PhD	3 (30)
Medical Specialisation in Geriatrics	2 (20)
**Professional profile**	
Advanced Practice nurse	3 (30)
Nurse Manager	2 (20)
Nurse Teacher	1 (10)
Research Nurse	2 (20)
Physician	1 (10)
Associate Professor	1 (10)

Abbreviation: CI, confidence interval, PhD: Doctor of Philosophy

#### UNCSD instrument development and consensus

**Fig. 2:**
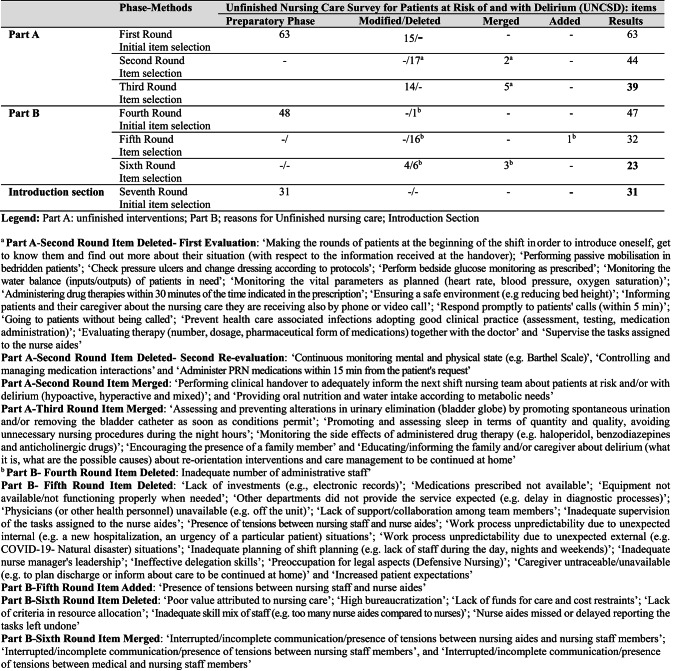
Development of the Unfinished Nursing Care Survey for Patients at Risk of and with Delirium (UNCSD), Delphi Study findings

Figure 2 illustrates the entire process of UNCSD development and consensus.

#### Part A

In the first round, 14 of the 63 interventions reported a mean score below 7 (indicating low importance). The interventions with the lowest scores were ‘Going to patients without being called’ (mean 5.9) and ‘Taking glucose measurements at the bedside as prescribed’ (mean 6.2). In contrast, 49 interventions reported a mean score above 7 (indicating greater importance for selection). The interventions with the highest scores were ‘Assess predisposing and precipitating risk factors for delirium (for hyper- or hypokinetic or mixed delirium) within the first 24 hours’ (mean 8.7); ‘Assessment of changes in vigilance, attention, cognitive and behavioral status within the first 24 hours and signs of marked change or fluctuation in attention, comprehension, or other cognitive behavioral functions’ (mean 8.6); and ‘Treatment of pain (administration of medication and non-pharmacological treatments)’ (mean 8.6).

In their notes, the panelists requested that two interventions be summarized: ‘Performing clinical handover to receive appropriate information about patients’ condition at the start of the shift’ and ‘Performing clinical handover to provide appropriate information about patients’ condition to the nursing team on the next shift.’

In the second round, the panelists first agreed to exclude the 14 interventions that were ranked below 7 (e.g., ‘Performing bedside glucose monitoring as prescribed’). They then agreed to group two interventions: ‘Performing clinical handover to adequately inform the next shift’s nursing team about at-risk patients and/or patients with delirium (hypoactive, hyperactive, and mixed)’ (mean 8; 80%) and ‘Providing oral nutrition and water intake according to metabolic needs’ (mean 9; 90%). In addition, panelistsre-evaluated the 49 interventions, and no intervention with a score below 50% was proposed for exclusion. Five interventions with a score of 50–69% were further evaluated (e.g., ‘Provide mouth care for patients who need it’), and all other interventions with scores above 70% were retained. Three interventions were excluded at the end of the second round (e.g., ‘Continuous monitoring of mental and physical condition [e.g., Barthel scale]’).

In the third round, 14 of the 44 interventions were modified (e.g., ‘Support, encouragement, and provision of walking aids according to the patient’s needs and problems’), and five were merged (e.g., ‘Assessing and prevention of changes in urinary elimination [bladder globe] by promoting spontaneous voiding and/or removal of bladder catheter as soon as conditions permit’).

All panelists agreed to the final version, which contains a total of 39 interventions in Part A (Fig. 2; Table 2, Supplementary Table 2).

**Table 2:** Part A unfinished nursing care survey for patients at risk of and with delirium (UNCSD)

Part A – Nursing Care interventions, Items
1. Continuous monitoring of precipitating risk factors with each change in the patient’s condition
2. Assessing the changes in vigilance, attention, and cognitive and behavioural status within the first 24 h after admission with instruments (e.g. 4 AT, CAM) or by clinical judgment
3. Performing physical/objective assessment of the patient (e.g. assessing the risk of pressure injury; signs and symptoms of infection at the insertion sites of devices)
4. Monitoring pain (verbal and non-verbal rating scales, e.g. PAINAID)
5. Treating pain by administering prescribed medication and using non-pharmacological techniques (e.g. relational, distraction)
6. Assessing the integrity, functioning and correct positioning of visual, hearing and dental prostheses
7. Assessing and preventing alterations in urinary elimination (bladder globe) by promoting spontaneous urination and/or removing the bladder catheter as soon as conditions permit
8. Promoting and assessing sleep in terms of quantity and quality, avoiding unnecessary nursing procedures during night hours
9. Assessing and preventing alterations in bowel elimination (diarrhoea and constipation)
10. Support, encouragement and provision of walking aids according to the patient’s needs and problems
11. Mobilising in a chair patient who need it
12. Providing mouth care to patients who need it
13. Providing personal hygiene for patients who need it
14. Helping to feed patients who are unable to feed themselves and/or have clinical problems (e.g. dysphagia)
15. Providing oral nutrition and water intake according to metabolic needs
16. Encouraging drinking and helping those who are unable to do so independently and/or have clinical problems
17. Encouraging the patient to maintain their autonomy or regain it
18. Minimising room/unit transfers
19. Ensuring patient comfort (e.g. microclimate, posture)
20. Minimising the negative effects of the hospital environment such as noise (bells, alarms, pumps, monitors) and lights (using indirect lights)
21. Ensuring the presence of objects for spatio-temporal orientation in the environment (e.g. clock, calendar, ‘where am I and where am I’ signs)
22. Encouraging the presence of personal objects (e.g. photos)
23. Encouraging the presence of a family member
24. Assessing the actual need to accommodate the person in a single room (Delirium Room)
25. Monitoring the side effects of administered drug therapy (e.g. haloperidol, benzodiazepines and anticholinergic drugs)
26. Assessing the effectiveness of care activities provided (e.g. visiting patients to ascertain that needs have been met)
27. Properly documenting the interventions provided and reviewing the care plan
28. Taking time and providing emotional support to patients and their family/caregivers by listening to their needs/concerns/feelings about delirium
29. Educating/informing the family and/or caregiver about delirium (what it is, what the possible causes are) about reorientation interventions and care management to be continued at home
30. Communicating effectively with the person: positioning myself in front of the person, calling him/her by name, specifying where he/she is, who I am, what my role is, the activities I am going to do (e.g. dressing, taking a blood sample), using consistent verbal and non-verbal language, with simple words and short sentences
31. Involving patients and caregivers in discharge planning
32. Monitoring more intensively by reassessing patients who are unstable or at risk of their condition deteriorating
33. Preventing negative outcomes for patients at risk (e.g. falls, pressure injuries, malnutrition)
34. Defining the personalised care plan and priorities for each patient trying to maintain the patient’s daily routine
35. Performing clinical handover to adequately inform the next shift nursing team about patients at risk of and/or with delirium (hypoactive, hyperactive and mixed)
36. Assessing the presence of predisposing risk factors (e.g. cognitive impairment, dementia, depression, advanced age, sensory deficits, severity of clinical condition, comorbidities, end of life) and precipitating risk factors for delirium (e.g. polypharmacotherapy, treatment with multiple psychoactive drugs, dehydration, malnutrition, hypoxia, immobility, pain, sleep and mood disorders) in the first 24 h after patient admission
37. Continuous monitoring alterations in vigilance, attention, cognitive and behavioural status with each change in patient condition and monitor the fluctuating course over time (hours or days) using instruments (e.g. 4 AT, CAM) or by clinical judgement
38. Working in teamwork, carrying out multi-professional interventions, performing multiple interventions together
39. Avoiding restraint (physical, pharmacological and environmental)

Abbreviations: 4AT=Assessment Test for Delirium & Cognitive Impairment; CAM= Confusion Assessment Method; PAINAD= Pain Assessment IN Advanced Dementia; *5-Point Likert Scale, from 1 “never” to 5 “always” unfinished

#### Part B

In the fourth round, members selected 47 out of 48 reasons for UNCSD. Of these, 27 reasons received an average score below 7 (indicating low importance); the lowest score was given to ‘Insufficient number of administrative staff’ (mean 4.5), which was therefore excluded. Additionally, 26 reasons received scores between 5 and 6.9 (e.g., ‘Increasing patient expectations,’ mean 6.2). Conversely, 21 reasons received an average score above 7; the highest scores were given to ‘Inappropriate environment (e.g., chaotic; high number of patients per room)’ (mean 8.2), ‘Repeated interruptions to nursing activities or continuity of care’ (mean 8.0), and ‘Insufficient number of nurses’ (mean 8.0).

In the fifth round, panelists rated their agreement (agreement score 7, 70%) with the inclusion of the 21 reasons (e.g., ‘Deficiencies in Education (e.g., incomplete training, lack of mentoring during transition from graduate to nurse)’) that had a mean score above 7. Panelists also excluded 16 reasons with a mean score below 5 (e.g., ‘Lack of investment (e.g., electronic records)’, ‘Prescribed medication not available’, ‘Equipment not available or not working properly when needed’, ‘Other departments did not perform as expected (e.g., delay in diagnostic processes)’) and retained those with mean scores between 5 and 6.9 on the GRADE scale (e.g., ‘Insufficient review of priorities during shift’). In this round, members also agreed to add the reason ‘Presence of tension between nursing staff and nursing assistants’ in Part B. The result of the fifth round was a list of 32 reasons.

In the final round, six reasons were excluded (scoring below 5; 50%) (e.g., ‘Low value placed on nursing care’, ‘High bureaucratization’); four were revised (e.g., ‘Lack of common procedures/protocols for high-risk patients and/or patients with delirium’, ‘Incomplete handover of care by staff on previous shift (e.g., on aspects concerning at-risk or delirium patients)’), and three were merged (e.g., ‘Interrupted/incomplete communication or presence of tension between nursing assistants and nursing staff’, ‘Interrupted/incomplete communication or presence of tension between nursing staff’, and ‘Interrupted/incomplete communication or presence of tension between medical and nursing staff’). Finally, panelists agreed to include 23 reasons in Part B of the UNCSD instrument (Fig. 2; Table 3, Supplementary Table 3).

**Table 3:** Part B unfinished nursing care survey for patients at risk of and with delirium (UNCSD)

Part B – Reasons for Unfinished Nursing Care, Items
1. Deficiencies in Education (e.g. incomplete education, in mentoring in the transition from graduate to nurse)
2. Ineffective performance of nurses (e.g. lack of experience, competence, culture, knowledge of the approach to patients with delirium)
3. Inadequate balance of nursing competences in the shift (e.g. too many new or inexperienced delirium nurses)
4. Incorrect allocation of priorities
5. Incomplete nursing handover by the staff of the previous shift (e.g. on aspects concerning patients at risk of/with delirium)
6. Poor time management and/or time optimisation skills
7. Insufficient number of nurses’ aides
8. Inadequate number of patients at risk of or with delirium assigned to each nurse
9. Increased nursing care needs of other patients (e.g. worsening clinical condition, complexity of care)
10. Insufficient number of nurses
11. High number of admissions/discharges during the shift
12. Interrupted/incomplete communication/presence of tensions between medical and nursing staff members
13. High staff turnover
14. Inadequate organisational model of nursing care delivery (e.g. task-based model)
15. Interrupted/incomplete communication/presence of tensions between nursing aides and nursing staff members
16. Interrupted/incomplete communication/presence of tensions between nursing staff members
17. Repeated interruptions of nursing activities and/or continuity of care
18. Inadequate environment (e.g. chaotic, large number of patients in each room)
19. Lack of shared procedures/protocols for patients at risk of and/or with delirium
20. Unexpected increase in the number of patients at risk of or with delirium in a critical condition
21. Inadequate planning of nursing care (e.g. activities to be performed simultaneously, unnecessary interventions)
22. Inadequate review of priorities during the shift
23. Inadequate attention to missed/delayed nursing care

* 4-Point Likert Scale, from 1 “not significant reason” to 4 “very significant reason”

### Introduction section

In the seventh round, panelists voted on the introduction section, which consisted of 26 questions derived from the UNCS tool [17] and eight questions proposed by researchers (see authors), reflecting the contribution of the available literature: three modified questions (e.g., the adequacy of education and training in prioritizing patients at risk of delirium or with delirium) and five new questions (e.g., the number of patients perceived to be at risk of delirium or with delirium during the last shift).

The introduction section proposed to the panelists therefore included: (a) demographic data; (b) education; (c) professional experience and profile; (d) available resources in the department where they were working as nurses at the time of the survey; (e) number of patients cared for during the last shift; (f) level of satisfaction with the role, profession, and group; and (g) intention to leave the department (no or yes, in the next six months or next 12 months). In addition, panelists voted on five questions introduced by the researchers regarding (1) the number of patients at risk of delirium during the last shift, (2) the number of patients with delirium during the last shift, (3) the model of care used (individualized care, no specific model, functional model of care), (4) the resources available to nurses during the last shift, and (5) the resources available to nursing assistants during the last shift. All panelists agreed, and the final version comprised 31 items (Fig. 2).

## Discussion

This Delphi study developed the UNCSD to systematically identify UNC, there by contributing to the prevention of delirium at the clinical level and ensuring appropriate interventions for patients with delirium, as well as identifying and addressing potential causes of UNC at the management level. In a seven-round Delphi process, consensus was reached among experts with multidisciplinary backgrounds, ensuring the clinical and methodological relevance of the new instrument [26]. The composition of the expert panel and their full participation in all Delphi rounds ensured a broad and qualified perspective. The panel represented all relevant areas in the field of delirium, including research, education, advanced clinical practice, and management [26], emphasizing the importance of a multidisciplinary and interprofessional approach to improving quality of care and communication [39].

The instrument consists of a general introduction and two sections, Part A and Part B, derived from the UNCS [23], the literature, and the opinions of the experts involved. Part A included additional interventions based on the results of a systematic review and the Nominal Group Technique [16], while Part B was based on a list of reasons identified through an integrative review [28]. The experts recognized that prevention and management for patients at risk of, and with, delirium require the implementation of multiple, integrated interventions to address the clinical and communication complexity of this condition [40].

In the introduction section, changes were made to the questions compared to the original UNCS instrument to improve understanding of Unfinished Nursing Care for patients at risk of, and with, delirium. For example, questions were added about the organizational model and the number of at-risk patients or patients with delirium under the nurse’s care [22]. These aspects should be considered antecedents of UNC, suggesting that patients at risk of, or with, delirium require personalized care models [41].

In Part A, the panelists selected 39 of 63 interventions considered essential for the care of patients with delirium, confirming the evidence in the literature on the need for multicomponent interventions as an effective non-pharmacological strategy for the prevention and management of delirium [42]. The interventions that received the highest scores in the first round emphasized the importance of early assessment of risk factors and cognitive status ‘Assessing the changes in vigilance, attention, cognitive and behavioural status within the first 24 hours and evidence of marked change or fluctuation in attention, comprehension, or other cognitive-behavioural functions’ [43], and pain management [44], highlighting the need for a timely and multidimensional approach [42]. Conversely, interventions that received an average score of less than 7 in the first round (e.g., ‘Going to patients without being called’; ‘Perform bedside glucose monitoring’) suggest that experts prioritise targeted and meaningful interventions for this population [45]. In the second round, the panelists used a structured consensus method [26], streamlining redundant interventions (for example, ‘Helping and encouraging patients who are unable to do so independently and/or have clinical problems to drink’ and ‘Motivating to take an oral nutritional and water intake according to their metabolic needs)’ and combining them into a single intervention: ‘Providing oral nutrition and water intake according to metabolic needs’. Interventions with inconsistent results were excluded (e.g., ‘Continuous monitoring of mental [e.g., orientation, short- and long-term memory, calculation, attention and concentration, object naming, command execution, writing, orientation in space and time, abstract thinking, judgment] and physical status [e.g., Barthel scale]’). In the third round, the panelists agreed on 39 interventions for Part A of the tool, representing preventive measures, non-pharmacological and pharmacological management strategies, and communication approaches for patients at risk of, or experiencing, delirium [2, 46].

In Part B, the expert panel selected 23 out of 48 items related to the reasons for UNC. The growing international interest in unfinished care – particularly regarding its underlying causes – demonstrates the complexity of this phenomenon, which is influenced by various factors, including human resources, increasing patient needs, and emotional aspects affecting nurses [47]. In the fourth round, panelists placed greater emphasis on structural factors (e.g., ‘Inadequate environment such as chaotic settings or high number of patients per room’) and organizational factors (e.g., ‘Repeated interruptions to nursing activities and/or continuity of care’), highlighting these as the most important perceived causes of UNC. In contrast, the item ‘Inadequate number of administrative staff’ was perceived as having less impact on direct care [13].

In the fifth round, the panel engaged in exclusion (e.g., ‘Lack of investments [e.g. electronic records]’), unification (e.g., ‘Interrupted/incomplete communication/presence of tensions between nursing staff members’), and agreement (e.g., ‘High number of admissions/discharges during the shift’) [26]. In the sixth round, the addition and merging of reasons related to disrupted communication and tension or conflict between professionals emphasized the importance of communication as a core competency within healthcare teams. This includes accountability, conflict management, decision-making processes, and coaching [48]. Communication breakdowns can affect continuity of care and increase the risk of omissions, jeopardizing patient safety [49].

Overall, the findings reflect the different levels suggested by Jones et al. [12], identifying reasons at the ward (e.g. ‘Inadequate number of patients at risk or with delirium assigned to each nurse’), patient (e.g., ‘Increased nursing care needs of other patients (e.g. worsening clinical condition, complexity of care)), and professional levels (e.g., ‘Poor time management and/or time optimisation skills’). Within this final category, the panel focused on the performance of nursing staff (e.g., ‘Ineffective performance of nursing staff (e.g. lack of experience, competence, culture, knowledge of managing patients with delirium)’). This reflects a broad understanding of performance that includes not only skills but also knowledge and cultural competence [50]. Nursing competencies related to delirium are a cornerstone of quality care. Timely and multidimensional clinical approaches [51], integration of delirium care, assessment of patient acuity, and training programs to improve nurses’ recognition and prioritization of delirium [52] are effective strategies. In this study, the panel identified these interventions and rationales as part of the development of an instrument for patients at risk of and with delirium, representing an initial phase of a validation study. Future perspectives may include incorporating the views of patients and family members, whose experiences could provide valuable insights and broaden the range of research topics. Another possible direction is to expand the panel to include a wider range of disciplines (e.g., physiotherapists), which could contribute to a more balanced representation of interventions and rationales.

### Limitations

Although the Delphi method is widely recognized as an effective tool for achieving expert consensus, this study has some limitations related to its design. First, the number of rounds – seven in total – exceeded the two or three usually recommended in the literature [26], reflecting the complexity of the topic and the challenges related to patients with or at risk of delirium, where the selection of interventions and their underlying reasons was particularly complex. Although the instrument considered care interventions deemed important for the prevention and management of patients with delirium, it is possible that some specific interventions, differentiated according to context, were not included [53]. Second, the way the Delphi process was conducted may have limited opportunities for in-depth and direct discussion among the experts, as only one round was conducted interactively online. To address this, participants in each round could enter detailed comments and suggestions in a blank field, and the interactive session was carefully moderated to ensure balanced participation and focused discussion. Third, the panel consisted of ten members selected based on their expertise and years of professional experience; however, no data were collected to better specify their competence in the field. It is also worth noting that each participant’s perspective was equally weighted throughout the modified Delphi process, and no expert knew the answers of the others. This approach was chosen to minimize peer pressure and encourage free and independent expression [35].

Fourth, although it was established a priori, the definition of the consensus threshold was influenced by the subjective opinions of the researchers involved, as is intrinsic to the nature of Delphi methods, which are based on the experience and judgment of experts [31]. Moreover, we used the GRADE Scale [32] to assign scores, but we did not apply a comprehensive assessment of content validity according to the standards proposed by the Consensus-based Standards for the Selection of Health Measurement Instruments (COSMIN) guidelines [54]. For this reason, future studies should integrate COSMIN procedures to ensure a more rigorous and comprehensive assessment. Finally, the analysis of the open-ended responses provided by participants in the different rounds was not conducted using a structured qualitative strategy. This recently proposed approach offers an innovative way to analyze open-ended responses in Delphi studies and could provide an opportunity to further explore the qualitative data [55].

## Conclusions

The Delphi study enabled the creation of the UNCSD instrument, which is divided into three parts: the introductory Sect. (31 items), Part A (39 items on the elements of the UNC), and Part B (23 items on the reasons), integrating evidence-based recommendations and the multidisciplinary expertise of the experts. The final version of the UNCSD instrument, which still needs validation, reflects the complexity of care for patients at risk of delirium and contributes to clinical practice by enabling the measurement of interventions to prevent and treat delirium, as well as understanding its causes. The instrument represents an important advancement in measuring UNC for patients at risk of delirium, supporting nurses’ decision-making in understanding the phenomenon and identifying UNC. Along with the underlying reasons, it enables nurses and their managers to define care priorities, balance demands, and ensure patient safety. Additionally, the list of interventions and reasons can serve as a checklist for newly hired nurses or inexperienced clinicians to address gaps in daily care, as older people at risk of or suffering from delirium are also at increased risk of receiving poor care. The instruments may also assist in research to identify reasons for variations in delirium occurrence across settings. Further studies are needed to validate the instruments and promote its use in different contexts, with the aim of improving the quality of care for patients at risk of delirium.

## Supplementary Information

Below is the link to the electronic supplementary material.

Supplementary Material 1

## Data Availability

No datasets were generated or analysed during the current study.
