# Supplementary material for: Measuring unfinished nursing care in patients with or at risk for delirium: a development and Delphi study
Source: Aging Clin Exp Res. 2026 Mar 10;38(1):97. doi: 10.1007/s40520-026-03332-4 (PMC13005806; doi:10.1007/s40520-026-03332-4)
Supplement: Supplementary file 1 — Supplementary Material 1 [file 40520_2026_3332_MOESM1_ESM.docx]

**Supplementary Table 1:** Checklist of the Conducting and REporting DElphi Studies (CREDES) guidelines for conducting and reporting Delphi studies [28]

| **Section / Topic** | **Item #** | **Recommendation** | **Reported on section** |
| --- | --- | --- | --- |
| **Purpose and rationale** | 1 | The purpose of the study should be clearly defined and demonstrate the appropriateness of the use of the Delphi technique as a method to achieve the research aim. A rationale for the choice of the Delphi technique as the most suitable method needs to be provided | Introduction |
| **Expert panel** | 2 | Criteria for the selection of experts and transparent information on recruitment of the expert panel, socio-demographic details including information on expertise regarding the topic in question, (non)response and response rates over the ongoing iterations should be  reported | Expert Panel |
| **Description of the methods** | 3 | The methods employed need to be comprehensible; this includes information on preparatory steps (How was available evidence on the topic in question synthesised, piloting of material and survey instruments, design of the survey instrument(s), the number and design of survey rounds, methods of data analysis, processing and synthesis of experts’ responses to inform the subsequent survey round and methodological decisions taken by the research team throughout the process | Methods  Study design  Preparatory Phase  Delphi Methods |
| **Procedure** | 4 | Flow chart to illustrate the stages of the Delphi process, including a preparatory phase, the actual ‘Delphi rounds’, interim steps of data  processing and analysis, and concluding steps | Phase  Delphi Methods  Figure 1 |
| **Definition and**  **attainment of consensus** | 5 | It needs to be comprehensible to the reader how consensus was achieved throughout the process, including strategies to deal with non- consensus | Consensus |
| **Results** | 6 | Reporting of results for each round separately is highly advisable in order to make the evolving of consensus over the rounds transparent. This includes figures showing the average group response, changes between rounds, as well as any modifications of the survey instrument  such as deletion, addition or modification of survey items based on previous rounds | Results |
| **Discussion of**  **limitations** | 7 | Reporting should include a critical reflection of potential limitations and their impact of the resulting guidance | Discussion  Limitations |
| **Adequacy of**  **conclusions** | 8 | The conclusions should adequately reflect the outcomes of the Delphi study with a view to the scope and applicability of the resulting  practice guidance | Conclusion |
| **Publication and dissemination** | 9 | The resulting guidance on good practice in palliative care should be clearly identifiable from the publication, including recommendations for transfer into practice and implementation. If the publication does not allow for a detailed presentation of either the resulting practice guidance or the methodological features of the applied Delphi technique, or both, reference to a more detailed presentation elsewhere should be made (e.g. availability of the full guideline from the authors or online; publication of a separate paper reporting on methodological details and particularities of the process (e.g. persistent disagreement and controversy on certain issues)). A dissemination plan should include endorsement of guidance by professional associations and health care authorities to facilitate implementation | NA |

Legend: NA: Not Available

**Supplementary Table 2:** Part A Unfinished Nursing Care Survey for Patients at Risk of and with Delirium (UNCSD)

|  | **Items, essential nursing interventions** | (Bassi et al [23]) | (Sist et al [16]) | **1 Round** | **2**  **Round** | **3**  **Round** |
| --- | --- | --- | --- | --- | --- | --- |
|  |  |  |  | Graded  Mean  Score ^a^ | Score Agreement  N (%)^b^  First Evaluation -/Second Re-evaluation | Score Agreement  N (%)^c^ |
| (1) | Performing clinical handover to receive adequate information on the patients' condition at the beginning of the shift | x |  | 8.4 | § |  |
| (63) | Perform clinical handover to adequately inform the next shift nursing team about patients' conditions | x |  | 8.3 | § |  |
| M (1-63) | Performing clinical handover to adequately inform the next shift nursing team about patients at risk and/or with delirium (hypoactive, hyperactive and mixed) |  |  | - | 8 (80)/- | 10 (100) |
| (2) | Making the rounds of patients at the beginning of the shift in order to introduce oneself, get to know them and find out more about their situation (with respect to the information received at the handover) | x |  | 6.8 | 0 (0) /- | - |
| (3) | Defining the personalised care plan and priorities for each patient (trying to maintain a daily routine for the person) | x | x | 7.4 | 9 (90) /- | - |
|  | Defining the personalised care plan and priorities for each patient trying to maintain the patient's daily routine |  |  | - | - | 10 (100) |
| (4) | Assisting and encouraging patients to walk and providing walking aids | x | x | 7.8 | 10 (100) /- | - |
|  | Support, encouragement and provision of walking aids according to the patient's needs and problems |  |  | - | - | 10 (100) |
| (5) | Mobilising in a chair patient who need it | x | x | 7.8 | 9 (90) /- | 10 (100) |
| (6) | Performing passive mobilisation in bedridden patients | x |  | 6.7 | 0 (0) /- | - |
| (7) | Helping patients who are unable to feed themselves and/or have clinical problems (e.g. dysphagia) to feed themselves | x |  | 7.5 | 7 (70) /- | - |
|  | Helping to feed patients who are unable to feed themselves and/or have clinical problems (e.g. dysphagia) |  |  | - | - | 10 (100) |
| (8) | Helping and encouraging patients who are unable to do so independently and/or have clinical problems to drink | x | x | 7.3 | (§) | - |
| (26) | Motivating to take an oral nutritional and water intake according to their metabolic needs (avoiding caffeine and heavy meals in the evening) |  | x | 6.9 | (§) | - |
| M (8-26) | Providing oral nutrition and water intake according to metabolic needs |  |  | - | 9 (90) /- | 10 (100) |
| (9) | Encourage the patient to maintain their autonomy or to recover it | x |  | 7.8 | 10 (100) /- | - |
|  | Encouraging the patient to maintain their autonomy or regain it |  |  | - | - | 10 (100) |
| (10) | Removing urinary catheter as soon as conditions permit and/or avoiding urinary catheterisation to encourage spontaneous urination |  | x | 7.8 | 8 (80) /- | (§) |
| (21) | Detecting issues in urinary elimination (presence of bladder globus) |  | x | 8.1 | 9 (90) /- | (§) |
| M (10-21) | Assessing and preventing alterations in urinary elimination (bladder globe) by promoting spontaneous urination and/or removing the bladder catheter as soon as conditions permit |  |  | - | - | 10 (100) |
| (11) | Provide personal hygiene for patients who need it | x |  | 7.9 | 8 (80) /- | - |
|  | Providing personal hygiene to patient who need it |  |  | - | - | 10 (100) |
| (12) | Provide mouth care to patients who need it | x |  | 7.6 | 6 (60) /10 (100) | - |
|  | Providing mouth care to patients who need it |  |  | - | - | 10 (100) |
| (13) | Perform physical assessment (e.g. skin integrity, invasive device insertion site) | x |  | 7.1 | 7 (70) /- | - |
|  | Performing physical/objective assessment of the patient (e.g. assessing the risk of pressure injury; signs and symptoms of infection at the insertion sites of the devices) |  |  | - | - | 10 (100) |
| (14) | Assessing predisposing and precipitating risk factors of delirium (for hyper- or hypokinetic or mixed delirium) within the first 24 hours |  | x | 8.7 | 10 (100) /- | - |
|  | Assessing the presence of predisposing risk factors (e.g. cognitive impairment, dementia, depression, advanced age, sensory deficits, severity of clinical condition, comorbidities, end of life) and precipitating risk factors for delirium (e.g. polypharmacotherapy, treatment with multiple psychoactive drugs, dehydration, malnutrition, hypoxia, immobility, pain, sleep and mood disorders) in the first 24 hours after patient admission |  |  | - | - | 10 (100) |
| (15) | Reassessing predisposing and precipitating risk factors of delirium (for hyper- or hypokinetic or mixed delirium) at each change (hours or days) |  | x | 7.6 | 9 (90) /- | - |
|  | Continuous monitoring of precipitating risk factors with each change in the patient's condition |  |  | - | - | 10 (100) |
| (16) | Assessing the changes in the vigilance, attention, cognitive and behavioural status within the first 24 hours and demonstration of a marked change or fluctuating course in attention, comprehension or other cognitive-behavioural functions |  | x | 8.6 | 10 (100) /- | - |
|  | Assessing the changes in vigilance, attention, cognitive and behavioural status within the first 24 hours after admission with instruments (e.g. 4 AT, CAM) or by clinical judgment |  |  | - | - | 10 (100) |
| (17) | Reassessing the changes in the vigilance, attention, cognitive and behavioural status at each change (hours or days) (e.g. with 4 AT scale) |  | x | 8.4 | 10 (100) /- | - |
|  | Continuous monitoring alterations in vigilance, attention, cognitive and behavioural status with each change in patient condition and monitor the fluctuating course over time (hours or days) using instruments (e.g. 4 AT, CAM) or by clinical judgement |  |  | - | - | 10 (100) |
| (18) | Continuous monitoring mental (e.g. orientation, short- and long-term memory, calculation, attention and concentration, object naming, command execution, writing, orientation in space and time, abstract reasoning, judgement) and physical state (e.g. Barthel Scale) |  | x | 7.8 | 5 (50) /0 (0) | - |
| (19) | Assessing the integrity, functioning and placing hearing, sight and dental prostheses |  | x | 8.4 | 8 (80) /- | - |
|  | Assessing the integrity, functioning and correct positioning of visual, hearing and dental prostheses |  |  | - | - | 10 (100) |
| (20) | Detecting issues in intestinal elimination (diarrhoea and constipation) |  | x | 7.9 | 9 (90) /- | - |
|  | Assessing and preventing alterations in bowel elimination (diarrhoea and constipation) |  |  | - | - | 10 (100) |
| (22) | Assessing sleep activity and patterns (Sist et al., 2022) |  | x | 7.9 | 9 (90) /- | (§) |
| (35) | Encouraging sleep by avoiding night-time procedures |  | x | 8.0 | 10 (100) /- | (§) |
| M (22-35) | Promoting and assessing sleep in terms of quantity and quality, avoiding unnecessary nursing procedures during the night hours |  |  | - | - | 10 (100) |
| (23) | Check pressure ulcers and change dressing according to protocols | x |  | 6.9 | 0 (0) /- | - |
| (24) | Perform bedside glucose monitoring as prescribed | x |  | 6.2 | 0 (0) /- | - |
| (25) | Monitoring the water balance (inputs/outputs) of patients in need | x |  | 6.5 | 0 (0) /- | - |
| (27) | Monitoring the vital parameters as planned (heart rate, blood pressure, oxygen saturation) | x | x | 6.9 | 0 (0) /- | - |
| (28) | Administering drug therapies within 30 minutes of the time indicated in the prescription | x |  | 6.3 | 0 (0) /- | - |
| (29) | Administer PRN medications within 15 min from the patient's request | x |  | 7.7 | 6 (60) /0 (0) | - |
| (30) | Monitor administered medications effects | x |  | 8.0 | 7 (70) /- | (§) |
| (32) | Administering and monitoring the effects of administered medication (e.g. haloperidol) |  | x | 7.9 | 9 (90) /- | (§) |
| M (30-32) | Monitoring the side effects of administered drug therapy (e.g. haloperidol, benzodiazepines and anticholinergic drugs) |  |  | - | - | 10 (100) |
| (31) | Controlling and managing medication interactions (Sist et al., 2022) |  | x | 7.1 | 6 (60) /0 (0) | - |
| (33) | Treating pain (administration of medication and non-pharmacological treatments) |  | x | 8.6 | 9 (90) /- | - |
|  | Treating pain by administering prescribed medication and using non-pharmacological techniques (e.g. relational, distraction) |  |  | - | - | 10 (100) |
| (34) | Ensure patients' comfort (microclimate, patient positioning) | x |  | 7.7 | 9 (90) /- | - |
|  | Ensuring patient comfort (e.g. microclimate, posture) |  |  | - | - | 10 (100) |
| (36) | Minimising the effects of the hospital environment such as noises (doorbell, alarms, pumps, monitors) and lights (avoiding direct light and using soft lights) |  | x | 8.0 | 9 (90) /- | - |
|  | Minimising of the negative effects of the hospital environment such as noise (bell, alarms, pumps, monitors) and lights (using indirect lights) |  |  | - | - | 10 (100) |
| (37) | Providing a clock, calendar and signs in the room (where they are and in which city) |  | x | 7.5 | 9 (90) /- | - |
|  | Ensuring the presence of objects for spatio-temporal orientation in the environment (e.g. clock, calendar, 'where am I and where am I' signs) |  |  | - | - | 10 (100) |
| (38) | Encouraging the presence of personal items (photos, bedspreads) |  | x | 7.2 | 9 (90) /- | - |
|  | Encouraging the presence of personal objects (e.g. photos) |  |  | - | - | 10 (100) |
| (39) | Ensuring a safe environment (e.g reducing bed height) |  | x | 6.7 | 0 (0) /- | - |
| (40) | Minimising the number of people in the room and placing the person in the single room (Delirium Room) |  | x | 7.5 | 9 (90) /- | - |
|  | Assessing the actual need to accommodate the person in a single room (Delirium Room) |  |  | - | - | 10 (100) |
| (41) | Minimising room/unit changes |  | x | 8.2 | 10 (100) /- | - |
|  | Minimising room/unit transfers |  |  | - | - | 10 (100) |
| (42) | Monitor pain as planned with verbal and non-verbal expression or using scales (e.g. PAINAID) | x | x | 8.4 | 10 (100) /- | - |
|  | Monitoring pain (verbal and non-verbal rating scales, e.g. PAINAID) |  |  | - | - | 10 (100) |
| (43) | Spend time with patients and their caregivers | x |  | 7.4 | 9 (90) /- | (§) |
| (46) | Supporting patients and carers emotionally by listening to their needs/concerns | x |  | 7.4 | 9 (90) /- | (§) |
| (47) | Encouraging the presence of the family and/or caregiver on a daily basis  and sharing the experience of delirium with the caregiver |  | x | 7.3 | 8 (80) /- | (§) |
| M (43-46-47) | Encouraging the presence of a family member |  |  | - | - | 10 (100) |
| (44) | Communicating with the person (calling him/her by name, explaining where I am, who I am, what my role is, what activities are taking place) with verbal and non-verbal language in a clear, simple way and position oneself in front of the person | x | x | 8.2 | 10 (100) /- | - |
|  | Communicating effectively with the person: positioning myself in front of the person, calling him/her by name, specifying where he/she is, who I am, what my role is, the activities I am going to do (e.g. dressing, taking a blood sample), using consistent verbal and non-verbal language, with simple words and short sentences |  |  | - | - | 10 (100) |
| (45) | Informing patients and their caregiver about the nursing care they are receiving also by phone or video call | x | x | 6.9 | 0 (0) /- | - |
| (48) | Involve patients and caregivers in the discharge planning | x |  | 7.5 | 8 (80) /- | - |
|  | Involving patients and caregivers in the discharge planning |  |  | - | - | 10 (100) |
| (49) | Teach patients and caregivers how to self-care at home | x |  | 7.1 | 7 (70) /- | (§) |
| (50) | Educating the family and/or caregiver. Contents: Risk factors and signs and symptoms of delirium, and changes in the person. Tools: Information leaflets) |  | x | 7.9 | 6 (60) /10(100) | (§) |
| (51) | Educating the family and/or caregivers. Contents: Re-orientation interventions for the person. Risk factors and signs and symptoms of delirium, and changes of the person Tools: Information leaflets |  | x | 7.7 | 10 (100) /- | (§) |
| M (49-50-51) | Educating/informing the family and/or caregiver about delirium (what it is, what are the possible causes) about re-orientation interventions and care management to be continued at home |  |  | - | - | 10 (100) |
| (52) | Respond promptly to patients' calls (within 5 min) | x |  | 6.3 | 0 (0) /- | - |
| (53) | Going to patients without being called (Bassi et al., 2020) | x |  | 5.9 | 0 (0) /- | - |
| (54) | Monitor more intensively by reassessing patients who are unstable or at risk of deteriorating condition | x |  | 7.7 | 8 (80) /- | - |
|  | Monitoring more intensively by reassessing patients who are unstable or at risk of deteriorating condition |  |  | - | - | 10 (100) |
| (55) | Prevent negative outcomes for patients at risk (e.g. falls, pressure ulcers, malnutrition) | x |  | 7.7 | 9 (90) /- | - |
|  | Preventing negative outcomes for patient at risk (e.g. falls, pressure injuries, malnutrition) |  |  | - | - | 10 (100) |
| (56) | Preventing restraints (physical, pharmacological, environmental and psychological or relational restraints) |  | x | 8.3 | 10 (100) /- | - |
|  | Avoiding restraint (physical, pharmacological and environmental) |  |  | - | - | 10 (100) |
| (57) | Prevent health care associated infections adopting good clinical practice (assessment, testing, medication administration) | x | x | 6.7 | 0 (0) /- | - |
| (58) | Offering the patient a multi-professional approach with multi-component interventions | x | x | 7.9 | 10 (100) /- | - |
|  | Working in teamwork, carrying out multi-professional interventions, performing multiple interventions together |  |  | - | - | 10 (100) |
| (59) | Evaluating therapy (number, dosage, pharmaceutical form of medications) together with the doctor |  | x | 6.7 | 0 (0) /- | - |
| (60) | Supervise the tasks assigned to the nurse aides | x |  | 6.3 | 0 (0) /- | - |
| (61) | Assess the effectiveness of the care provided, for example reviewing if nursing care needs have been met | x |  | 7.4 | 9 (90) /- | - |
|  | Assessing the effectiveness of care activities provided (e.g. visiting patients to ascertain that needs have been met) |  |  | - | - | 10 (100) |
| (62) | Document properly the interventions provided and the revision of the care plan | x |  | 7.2 | 8 (80) /- | - |
|  | Properly documenting the interventions provided and reviewing the care plan |  |  | - | - | 10 (100) |

^a^ Graded from 1 (least important) to 9 (most important); Score Agreement for inclusion of items in the scale <5 excluded; 5-6.9 subject to further evaluation; >7 considered

^b^ Score Agreement to include items within the scale <50% excluded; 50-69 % subject to further evaluation; >70% considered

^c^ Score Agreement to include items within the scale >90% considered

**Abbreviation:** M: Merged; PAINAD: Pain Assessment IN Advanced Dementia; PRN; pro re nata (as needed); 4AT: Assessment test for delirium & cognitive impairment

(§) Commentary on revaluation and merging items

**Supplementary Table 3:** Part B Unfinished Nursing Care Survey for Patients at Risk of and with Delirium (UNCSD)

|  | **Items, Unfinished nursing care reasons** | (Bassi et al. [23]) | (Sist et al. [29]) | **4 Round** | **5**  **Round** | | **6**  **Round** |
| --- | --- | --- | --- | --- | --- | --- | --- |
|  |  |  |  | Graded  Mean^a^ | Graded  Mean^b^ | Score Agreement  N(%)^c^ | Score Agreement  N(%)^d^ |
| (1) | Poor value attributed to nursing care |  | x | 6.8 | 5.2 | - | 3 (30) |
| (2) | Inadequate attention to missed/delayed nursing care |  | x | 6.4 | 5.7 | - | 9 (90) |
| (3) | High bureaucratisation |  | x | 7.3 |  | 7 (70) | 2 (20) |
| (4) | Lack of investments (e-g electronic records) |  | x | 6.3 | 2.7 | - |  |
| (5) | Lack of funds for care and cost restraints |  | x | 7.7 | - | 7 (70) | 2 (20) |
| (6) | Inadequate environment (e.g. chaotic, large number of patients in each room) |  | x | 8.2 | - | 7 (70) | 10 (100) |
| (7) | Lack of criteria in resource allocation |  | x | 7.2 | - | 7 (70) | 4 (40) |
| (8) | High staff turnover |  | x | 7.6 | - | 7 (70) | 9 (90) |
| (9) | Medications prescribed not available | x |  | 5.1 | 4.0 | - | - |
| (10) | Equipment not available/not functioning properly when needed | x |  | 5.8 | 3.7 | - | - |
| (11) | Other departments did not provide the service expected (e.g. delay in diagnostic processes) | x |  | 5.3 | 4.5 | - | - |
| (12) | Inadequate number of patients at risk or with delirium assigned to each nurse | x | x | 7.7 | - | 7 (70) | 10 (100) |
| (13) | Insufficient number of nurses | x |  | 8.0 | - | 7 (70) | 10 (100) |
| (14) | Insufficient number of nurses’ aides | x |  | 8.0 | - | 7 (70) | 10 (100) |
| (15) | Inadequate number of administrative staff | x |  | 4.5 | - | - | - |
| (16) | Inadequate skill mix of staff (e.g. too many nurse aides compared to nurses) | x |  | 6.0 | 5.6 | - | 4 (40) |
| (17) | Physicians (or other health personnel) unavailable (e.g. off the unit) |  | x | 6.2 | 4.5 | - | - |
| (18) | Lack of support/collaboration among team members | x |  | 6.4 | 4.9 | - | - |
| (19) | Nurse aides missed or delayed to report the tasks left undone | x |  | 6.0 | 5.0 | - | 3 (30) |
| (20) | Inadequate supervision of the tasks assigned to the nurse aides | x |  | 6.8 | 4.8 | - | - |
| (21) | Presence of tensions between nursing staff and nurse aides | x |  | 6.6 | 4.9 | § | § |
| (22) | Incomplete or interrupted communication between nursing staff and nurse aides/assistive personnel | x |  | 7.3 | - | 7 (70) | § |
| M (21-22) | Interrupted/incomplete communication/presence of tensions between nursing aides and nursing staff members |  |  | - | - | - | 10 (100) |
| (23) | Tension/conflicts within the nursing staff | x |  | 6.5 | 5.0 | § |  |
| (24) | Incomplete or interrupted communication among nursing staff | x |  | 6.9 | 5.5 | § |  |
| M (23-24) | Interrupted /incomplete communication/presence of tensions between nursing staff members |  |  | - | - | - | 10 (100) |
| (25) | Tension/conflicts between nursing and medical staff | x |  | 7.0 | - | 7 (70) | § |
| (26) | Incomplete or interrupted communication between nursing and medical staff | x |  | 7.4 | - | 7 (70) | § |
| M (25-26) | Interrupted /incomplete communication/presence of tensions between medical and nursing staff members |  |  | - | - | - | 10 (100) |
| (27) | Work process unpredictability due to unexpected internal (e.g. a new hospitalization, an urgency of a particular patient) situations |  | x | 5.8 | 4.4 | - | - |
| (28) | Work process unpredictability due to unexpected external (e.g. COVID-19- Natural disaster) situations |  | x | 5.0 | 3.4 | - | - |
| (29) | High number of admissions/discharges during the shift | x |  | 7.9 | - | 7 (70) | 10 (100) |
| (30) | Lack of shared procedures for the patient at risk and/or with delirium |  | x | 6.8 | 5.5 | - | - |
|  | Lack of shared procedures/protocols for the patient at risk and/or with delirium |  |  | - | - | - | 10 (100) |
| (31) | Repeated interruptions of nursing activities or/and continuity of care | x | x | 8.0 | - | 7 (70) | 10 (100) |
| (32) | Inadequate planning of shift planning (e.g. lack of staff during the day, nights and weekends) |  | x | 6.9 | 4.9 | - | - |
| (33) | Inadequate planning of nursing care (e.g. activities to be performed simultaneously, unnecessary interventions) |  | x | 7.1 |  | 7 (70) | 10 (100) |
| (34) | Inadequate review of priorities during the shift | x |  | 6.6 | 6.4 | - | 9 (90) |
| (35) | Incorrect allocation of priorities | x |  | 6.8 | 5.5 | - | 9(90) |
| (36) | Inadequate organisational model of nursing care delivery (e.g. task-based model) |  | x | 7.3 | - | 7 (70) | 10 (100) |
| (37) | Incomplete nursing handover by the staff of the previous shift | x |  | 7.4 | - | 7 (70) | - |
|  | Incomplete nursing handover by the staff of the previous shift (e.g. on aspects concerning patients at risk/with delirium) |  |  | - | - | - | 10 (100) |
| (38) | Inadequate nurse manager's leadership |  | x | 5.4 | 4.7 | - | - |
| (39) | Inadequate balance of nursing competences in the shift (e.g. too many new or inexperienced delirium nurses) | x |  | 7.2 | - | 7 (70) | 10 (100) |
| (40) | Poor time management and/or time optimisation skills |  | x | 7.1 | - | 7 (70) | 10 (100) |
| (41) | Ineffective performance of nurses (e.g. lack of experience, competence, knowledge on the approach to the patient with delirium) |  | x | 6.8 | 7.0 | - | - |
|  | Ineffective performance of nurses (e.g. lack of experience, competence, culture, knowledge of the approach to the patient with delirium) |  |  | - | - | - | 10 (100) |
| (42) | Ineffective delegation skills |  | x | 6.8 | 3.7 | - | - |
| (43) | Deficiencies in Education (e.g. incomplete education, in mentoring in the transition from graduate to nurse) |  | x | 7.4 | - | 7 (70) | 10 (100) |
| (44) | Preoccupation for legal aspects (Defensive Nursing) |  | x | 5.3 | 2.9 | - | - |
| (45) | Unexpected increase in the number of patients at risk or with delirium in critical condition | x | x | 7.5 | - | 7 (70) | 10 (100) |
| (46) | Caregiver untraceable/unavailable (e.g. to plan discharge or inform about care to be continued at home) | x |  | 6.7 | 4.5 | - |  |
| (47) | Increased nursing care needs of other patients (e.g. worsening clinical condition, complexity of care, cognitive disorders) |  | x | 7.9 | - | 7 (70) | - |
|  | Increased nursing care needs of other patients (e.g. worsening clinical condition, complexity of care) |  |  | - | - | - | 10 (100) |
| (48) | Increased patient expectations |  | x | 6.2 | 2.5 | - | - |

^a^ Graded from 1 (least important) to 9 (most important). Score Agreement for inclusion of items in the scale <5 excluded; 5-6.9 subject to further evaluation; >7 considered

^b^ Score Agreement for inclusion of items in the scale <5 excluded; 5-6.9 subject to further evaluation; >7 considered

^c^ Score above 7 rated; Score Agreement to include items within the scale <50% excluded; 50-69 % subject to further evaluation; >70% considered

^d^ Score Agreement to include items within the scale >90% considered

(§) Commentary on revaluation and merging items

**Abbreviation:** M: Merged COVID-19: Coronavirus Disease-19
